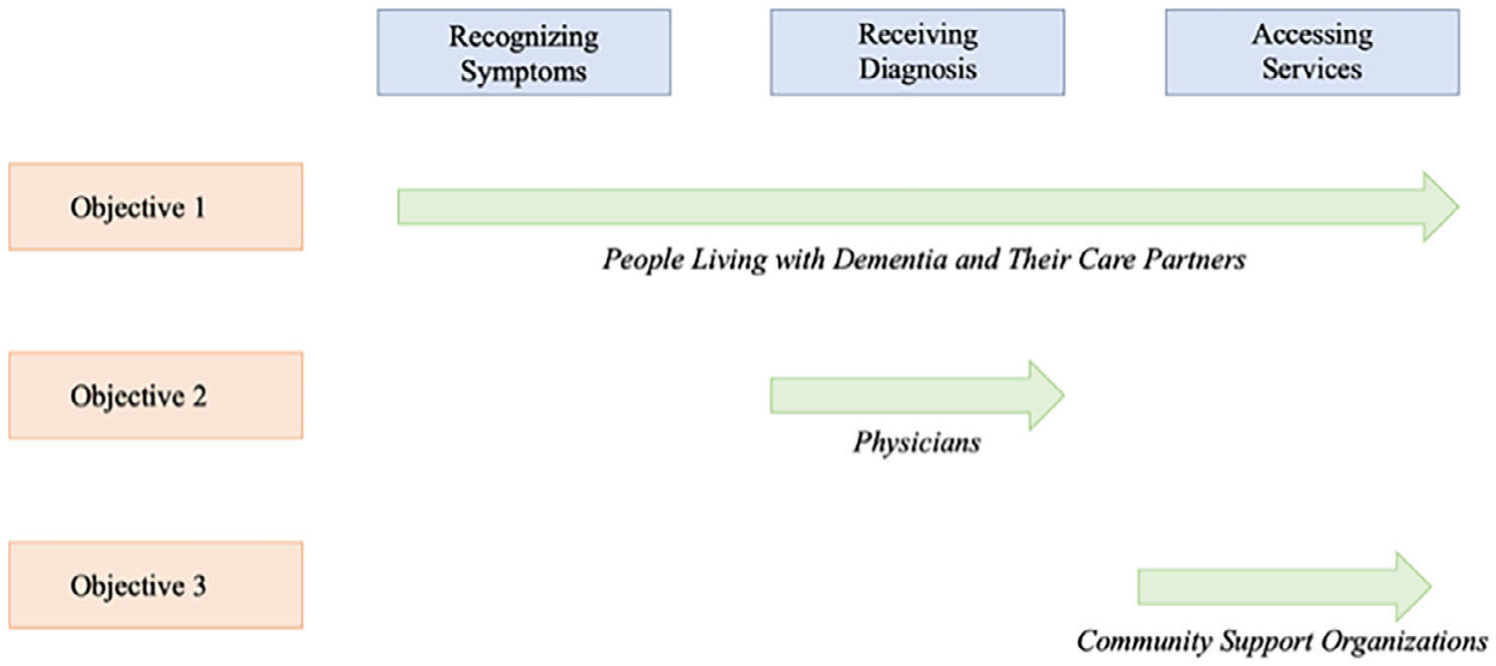# Dementia Dastan: Understanding the Experiences of South Asian Canadians Living with Dementia and their Care Partners

**DOI:** 10.1002/alz70858_099141

**Published:** 2025-12-25

**Authors:** Navjot Gill‐Chawla, George Heckman, Carrie A McAiney, Catherine Tong

**Affiliations:** ^1^ University of Waterloo, Waterloo, ON, Canada; ^2^ Alzheimer Society of Alberta and Northwest Territories, Edmonton, AB, Canada; ^3^ Western University, London, ON, Canada; ^4^ Schlegel‐UW Research Institute for Aging, Waterloo, ON, Canada

## Abstract

**Background:**

Dementia care in Canada must address the unique needs of South Asian Canadians, a growing population facing cultural and systemic barriers. Cultural stigma, language challenges, and a lack of culturally appropriate resources delay dementia recognition, diagnosis, and access to services. These barriers burden care partners, who navigate caregiving within cultural expectations and limited formal support. While some experiences of individuals living with dementia and their care partners are shared across communities, South Asian Canadians face additional challenges, including stigma rooted in cultural beliefs and limited access to culturally aligned services. Despite strong caregiving traditions, there is limited research on shared and culturally specific aspects of their experiences. This research examines the experiences of individuals with dementia, their care partners, physicians diagnosing dementia, and community support organization employees to identify barriers, strengths, and strategies for improving culturally inclusive dementia care.

**Method:**

A qualitative, interpretive phenomenological approach was used through three interconnected studies conducted in Alberta, British Columbia, and Ontario. Study 1 examines the experiences of 16 participants (14 care partners and two individuals living with dementia) across stages of recognizing symptoms, obtaining a diagnosis, and accessing services. Study 2 investigates the perspectives of 13 physicians on diagnosing dementia in South Asian Canadians. Study 3 captures the insights of 14 employees from community support organizations providing dementia services. Semi‐structured interviews were conducted in English, Hindi, and Punjabi, and reflexive thematic analysis was applied to identify recurring and distinct themes.

**Result:**

Study 1 found barriers to recognizing dementia, challenges in obtaining a diagnosis, and difficulties in accessing services post‐diagnosis, which were exacerbated by cultural beliefs, stigma, and unfamiliarity with healthcare systems. Study 2 revealed barriers to diagnosis, cultural and generational influences, and language challenges. Study 3 emphasized cultural sensitivity, trust‐building, and the importance of partnerships with cultural organizations while highlighting systemic funding gaps.

**Conclusion:**

This research demonstrates the need for cultural humility in dementia care practices and policies. Findings underscore the importance of early diagnosis, community engagement, and developing culturally tailored resources to support families. Addressing systemic barriers and increasing funding for culturally sensitive services is essential to providing equitable dementia care for Canada's diverse population.